# Effects of L-arginine, guanidinoacetic acid and L-citrulline supplementation in reduced-protein diets on bone morphology and mineralization of laying hens

**DOI:** 10.1016/j.aninu.2023.03.012

**Published:** 2023-06-16

**Authors:** Hiep Thi Dao, Amy F. Moss, Emma J. Bradbury, Robert A. Swick

**Affiliations:** a School of Environmental and Rural Science, Faculty of Science, Agriculture, Business and Law, University of New England, Armidale, New South Wales, 2351, Australia; b Faculty of Animal Science, Vietnam National University of Agriculture, Trau Quy Town, Gia Lam District, Hanoi, 100000, Vietnam; c Baiada Poultry Pty Limited, Pendle Hill, New South Wales, 2145, Australia

**Keywords:** Ash, Breaking strength, Femur, Low protein, Minerals, Digestibility

## Abstract

The alterations in feed ingredients and the nutrient matrix to produce reduced-protein diets may affect bone morphology and mineralization in laying hens. This study was implemented to determine the effects of L-arginine (Arg), guanidinoacetic acid (GAA), and L-citrulline (Cit) supplementation to Arg-deficient reduced-protein diets on bone morphology, strength, and mineralization status of laying hens. Individually housed Hy-Line Brown laying hens were evenly distributed to five dietary treatments with 25 replicates per treatment from 20 to 40 wk of age. Treatments consisted of a standard protein diet (17% crude protein, SP), a reduced-protein diet deficient in Arg (13% crude protein, RP), and RP supplemented with Arg (0.35% Arg, RP-Arg), GAA (0.46% GAA equivalent to 0.35% Arg, RP-GAA), or Cit (0.35% Cit equivalent to 0.35% Arg, RP-Cit) to reach the Arg level of SP diets. Birds fed the SP diet had similar bone weight, ash, length, width, Seedor index, breaking strength, and serum mineral concentration, but higher toe B level (*P* < 0.001) compared to those fed the RP diet at wk 40. Birds fed the SP diet consumed more but also excreted more K and B compared to those fed the RP diet (*P* < 0.01). Birds fed the SP diet had lower Cu digestibility (*P* = 0.01) and higher B retention (*P* < 0.01) compared to those offered the RP diet. Supplementation of Arg, GAA, and Cit to the RP diet increased relative femur weight and length (*P* < 0.001). Citrulline supplementation also increased relative tibia and femur ash, and Zn digestibility (*P* < 0.05). Supplementation of GAA to the RP diet decreased serum Ca, P, and Mg levels, decreased tibia Fe and Mg levels and toe Mg level, but increased Al, Fe, Zn, and Mn digestibility (*P* < 0.05). The current findings demonstrated the capacity of laying hens to adapt to low mineral intake by increasing mineral utilization. Overall, bone morphology and breaking strength, and serum mineral level in laying hens were not influenced by dietary CP levels. Dietary Arg, GAA, or Cit supplementation were effective in improving bone morphology and mineralization in laying hens fed Arg-deficient RP diets.

## 1 Introduction

Reducing crude protein (CP) levels in poultry diets has received increasing interest due to improved protein digestibility and litter quality, reduced water intake, and reduced ammonia production (Hilliar et al., 2020). Various studies investigate the effects of feeding reduced-protein diets on production performance and nitrogen digestibility in broilers and laying hens (Belloir et al., 2017; Chrystal et al., 2020a; Hilliar et al., 2019; Sun et al., 2022; Zhou et al., 2021). However, research on the effects of feeding reduced-protein diets on bone morphology and mineralization status in birds is often overlooked. The alterations in feed ingredients and nutrient matrix to produce reduced-protein diets, namely the higher inclusion of cereal grains at the expense of soybean meal, may affect the bone morphology and mineralization in birds compared to those offered the standard protein diets, given that soybean meal contains higher levels of available P and K compared to cereal grains (Chrystal et al., 2020b; Cowieson et al., 2020; Eeckhout and De Paepe, 1994; Weremko et al., 1997).

Higher inclusions of dietary fiber (non-digestible carbohydrates) have been shown to increase intestinal mineral absorption, particularly Ca and Fe, in laying hens. This might be attributed to the increased short-chain fatty acid production following increased microbial fermentation of non-digestible carbohydrates in the gut (Gultemirian et al., 2014). It has been suggested that short-chain fatty acid production may increase mineral solubility and bioavailability through reducing intestinal pH and assist in transporting the minerals from the intestine to blood (Bar, 2009; Gultemirian et al., 2014). On the other hand, there is evidence that increasing dietary fat level increased serum P level and tended to increase tibia P level in laying hens (Usayran et al., 2001). Thus, it is sensible that the alterations in dietary fiber and lipid content in the formulation of reduced-protein diets may influence mineral metabolism and utilization in birds. A study by Yalcin et al. (1998) indicated that tibia weight, length, diameter, radiographic density, and breaking strength were not affected by dietary CP level in broilers. More recently, Dao et al. (2022) reported increased mineral digestibility in broilers fed reduced-protein diets compared to those fed standard protein diets. Nevertheless, bone morphology and mineralization following dietary CP reduction may differ in laying hens due to the differences in production function between broilers and laying hens such as the use of bone to store minerals in laying hens to later meet the high mineral demands of producing eggshell. Laying hens mobilize Ca from bones to produce eggshells if the dietary Ca level is insufficient (Świątkiewicz et al., 2010). Also, it was previously reported that higher toe weight, tibia ash content, and P retention were observed in broiler chickens supplemented with 250 mg/kg Cu from Cu lysinate compared to those fed Cu sulfate; whereas, feeding these sources of Cu had no effects in laying hens (Banks et al., 2004). However, this information is largely unknown.

Arginine (Arg) has been found to be important for bone development through its engagement in collagen and connective tissue formation (Corzo et al., 2003; Jahanian, 2009). Previous studies have reported decreased bone mineral density in broilers and laying hens fed diets deficient in Arg (Castro et al., 2019a, 2019b, 2020). Guanidinoacetic acid (GAA), a precursor of creatine, has been demonstrated to possess an Arg-sparing effect in birds (Dao et al., 2021a; DeGroot et al., 2018). Furthermore, dietary GAA supplementation might increase tibia density and breaking strength in broilers (Khajali et al., 2020). Similarly, the Arg-sparing effects of citrulline (Cit) have been demonstrated in birds (Dao et al., 2021a; Su and Austic, 1999) where Cit is converted to Arg in the kidney and other extrahepatic tissues (Fernandes and Murakami, 2010; Tamir and Ratner, 1963).

A study was implemented to investigate the effects of Arg, GAA, and Cit supplementation in an Arg-deficient reduced-protein diet in laying hens. The results on laying performance, egg quality, and nutrient digestibility were reported in Dao et al. (2021b). The results showed that hens receiving the standard protein diet had higher egg mass, egg weight, and shell weight compared to those fed the reduced-protein diet deficient in Arg with or without Arg, GAA and Cit supplementation (Dao et al., 2021b). Supplementation of either Arg or Cit to the Arg-deficient reduced-protein diet did not affect egg mass, egg weight, shell weight, shell thickness, and shell breaking strength whereas GAA supplementation decreased egg mass (Dao et al., 2021b). Additionally, it was hypothesized that bone morphological parameters and mineral concentration, and mineral digestibility would decrease with the Arg-deficient reduced-protein diet and the supplementation of Arg, GAA, or Cit to the reduced-protein diet would restore bone quality and mineral concentration. The effects of Arg, GAA, and Cit supplementation in an Arg-deficient reduced-protein diet on bone morphology, strength, and mineralization status of laying hens were reported in this paper.

## 2 Materials and methods

### 2.1 Animal ethics statement

Experimental procedures were approved by the University of New England's Animal Ethics Committee and met the requirements of the Australian code of practice for care and use of animals for scientific purposes (NHMRC, 2013). This study was performed in accordance and full compliance with the approved guidelines and regulations. The study reported in this paper complies with the ARRIVE guidelines.

### 2.2 Experimental design, diets, and data collection

Details on the management, experimental design, dietary treatments, data collection, and feed analysis are presented in Dao et al. (2021b). This study was implemented at the University of New England Layer Cage Facility in Armidale, NSW, Australia. Individually housed Hy-line Brown laying hens (*n* = 125) were evenly distributed by average body weight to five dietary treatments with 25 replicate cages (30 cm × 50 cm × 45 cm, width × depth × height) per treatment and production parameters measured from 20 to 40 wk. Birds were raised in a curtain-sided house. Each cage was equipped with one nipple drinker and one feeder. The lighting program of 16 h light:8 h dark was applied throughout the study. The dietary treatments were: a standard protein diet (17% crude protein, SP), a reduced-protein diet deficient in Arg (13% crude protein, RP), and RP supplemented with Arg (0.35% Arg, RP-Arg), GAA (0.46% GAA equivalent to 0.35% Arg, RP-GAA) or Cit (0.35% Cit, RP-Cit) to the Arg level of SP. Diets were provided as mash and fed ad libitum to the birds throughout the study. Levels of essential amino acids were chosen according to Hy-Line Brown nutritional recommendation for the laying period (Hy-Line International, 2016). Levels of supplemental Cit in the RP-Cit diet were equivalent to supplemental Arg level in the RP-Arg diet on a molar basis whereas GAA level in the RP-GAA diet was selected based on the previous finding of Ringel et al. (2013) that GAA has 77% Arg equivalence for feed conversion. Detailed information on the composition and nutrient content of the diets is presented in Table 1, Table 2. These tables were also reported in Dao et al. (2021b). At wk 40, blood serum, tibia, femur, and toe (from the right leg) were collected from 10 hens per treatment for measurements of mineral composition and bone morphology. At wk 40, also, 6 hens per treatment with body weights (BW) close to the average BW of the treatments were chosen for determination of mineral digestibility using the total collection of excreta method over 5 consecutive days.

**Table 1:** Diet composition of experimental treatments (%, as-is basis).

Item	SP^1^	RP^2^	RP-Arg^3^	RP-GAA^3^	RP-Cit^3^
Ingredients					
Wheat	19.71	34.41	34.06	34.06	34.06
Sorghum	30.00	30.00	30.00	30.00	30.00
Soybean meal	13.28	–	–	–	–
Canola meal	8.00	8.00	8.00	8.00	8.00
Barley	5.00	8.00	8.00	8.00	8.00
Wheat millrun	8.57	5.00	5.00	5.00	5.00
Canola oil	2.99	0.69	0.69	0.69	0.69
Limestone	9.50	9.84	9.84	9.84	9.84
Dicalcium phosphate	1.78	1.86	1.86	1.86	1.86
Salt	0.28	0.28	0.28	0.28	0.28
Sodium bicarbonate	0.10	0.10	0.10	0.10	0.10
Xylanase^4^	0.01	0.01	0.01	0.01	0.01
Vitamin-mineral premix^5^	0.10	0.10	0.10	0.10	0.10
Choline chloride (60%)	0.22	0.27	0.27	0.27	0.27
L-Lys	0.14	0.53	0.53	0.53	0.53
D,L-Met	0.21	0.28	0.28	0.28	0.28
L-Thr	0.09	0.25	0.25	0.25	0.25
L-Trp	–	0.02	0.02	0.02	0.02
L-Ile	–	0.20	0.20	0.20	0.20
L-Arg	–	–	0.35	–	–
GAA	–	–	–	0.46	–
L-Cit	–	–	–	–	0.35
L-Val	–	0.16	0.16	0.16	0.16
Pigment jabiru red	0.004	0.004	0.004	0.004	0.004
Pigment jabiru yellow	0.003	0.003	0.003	0.003	0.003
Calculated composition					
Dry matter	91.04	91.34	91.34	91.34	91.34
AMEn^6^, kcal/kg	2750	2750	2750	2750	2750
CP	17.00	13.00	13.50	14.00	13.50
Crude fat	6.20	3.96	3.96	3.96	3.96
Ash	4.81	4.12	4.12	4.12	4.12
GAA	0.00	0.00	0.00	0.46	0.00
L-Cit	0.00	0.00	0.00	0.00	0.35
Dig.^7^ Arg	0.90	0.54	0.89	0.54	0.54
Dig. Lys	0.76	0.76	0.76	0.76	0.76
Dig. Met	0.44	0.46	0.46	0.46	0.46
Dig. Cys	0.25	0.22	0.22	0.22	0.22
Dig. Met + Cys	0.67	0.67	0.67	0.67	0.67
Dig. Trp	0.19	0.15	0.15	0.15	0.15
Dig. Ile	0.59	0.57	0.57	0.57	0.57
Dig. Thr	0.55	0.55	0.55	0.55	0.55
Dig. Val	0.67	0.67	0.67	0.67	0.67
Dig. Gly	0.50	0.34	0.34	0.34	0.34
Calcium	4.10	4.22	4.22	4.22	4.22
Available phosphorus	0.46	0.46	0.46	0.46	0.46
Sodium	0.18	0.18	0.18	0.18	0.18
Potassium	0.64	0.42	0.42	0.42	0.42
Chloride	0.28	0.36	0.36	0.36	0.36
Choline, mg/kg	2,000	2,000	2,000	2,000	2,000
Linoleic acid	2.10	1.57	1.57	1.57	1.57
DEB^8^, mEq/kg	166	118	118	118	118

GAA = guanidinoacetic acid; Cit = citrulline; CP = crude protein.

1 SP: standard protein diet with 17% CP.

2 RP: reduced protein diet with 13% CP.

3 L-Arg, GAA and L-Cit were added on top of the RP diets at 0.35%, 0.46% and 0.35% in diets, RP-Arg, RP-GAA and RP-Cit, respectively, to the level of Arg in SP.

4 Xylanase: Econase XT-25, AB Vista.

5 Vitamin-mineral premix provided the following per kilogram of vitamin-mineral premix: vitamin A, 10 MIU; vitamin D, 3 MIU; vitamin E, 20 g; vitamin K, 3 g; nicotinic acid, 35 g; pantothenic acid, 12 g; folic acid, 1 g; riboflavin, 6 g; cyanocobalamin, 0.02 g; biotin, 0.1 g; pyridoxine, 5 g; thiamine, 2 g; copper, 8 g as copper sulphate pentahydrate; cobalt, 0.2 g as cobalt sulphate (21%); molybdenum, 0.5 g as sodium molybdate; iodine, 1 g as potassium iodide (68%); selenium, 0.3 g as selenium (2%); iron, 60 g as iron (30%); zinc, 60 g as zinc sulphate (35%); manganese, 90 g as manganous oxide (60%); antioxidant, 20 g.

6 AMEn: nitrogen-corrected apparent metabolizable energy.

7 Digestible amino acid coefficients for raw ingredients were determined by Near-Infra Red spectroscopy (Foss NIR 6500, Denmark) standardized with Evonik AMINONIR Advanced calibration.

8 Dietary electrolyte balance (DEB) was calculated as 10,000 × (Na^+^ + K^+^ – Cl^−^).

**Table 2:** Analyzed nutrient values of experimental diets (%, as-is basis).^1^

Nutrient composition	SP^2^		RP^3^		RP-Arg^4^		RP-GAA^4^		RP-Cit^4^	
Dry matter	92.64	-	92.94	-	92.60	-	92.55	-	92.64	-
Gross energy, kcal/kg	3,734	-	3,446	-	3,559	-	3,495	-	3,523	-
CP	17.24	-	13.08	-	13.50	-	14.16	-	13.69	-
Crude fat	5.21	-	2.97	-	2.97	-	2.97	-	2.97	-
Crude fiber	5.32	-	9.70	-	9.70	-	9.70	-	9.70	-
Ash	17.08	-	16.15	-	14.67	-	16.46	-	15.36	-
Calcium	5.33	-	6.35	-	5.51	-	6.05	-	5.78	-
Total phosphorus	0.82	-	0.77	-	0.77	-	0.79	-	0.77	-
GAA	0.00	-	0.00	-	0.00	-	0.55	-	0.00	-
Cit	0.00	-	0.00	-	0.00	-	0.00	-	0.35	-
Arg	1.00	(1.06)	0.59	(0.64)	0.93	(0.99)	0.59	(0.64)	0.59	(0.64)
Lys	0.99	(0.88)	1.02	(0.84)	1.02	(0.84)	1.02	(0.84)	1.02	(0.84)
Met	0.46	(0.48)	0.52	(0.49)	0.52	(0.49)	0.52	(0.49)	0.52	(0.49)
Cys	0.32	(0.31)	0.26	(0.26)	0.26	(0.26)	0.26	(0.26)	0.26	(0.26)
Met + Cys	0.78	(0.75)	0.78	(0.73)	0.78	(0.73)	0.78	(0.73)	0.78	(0.73)
Trp	0.24	(0.22)	0.19	(0.17)	0.19	(0.17)	0.19	(0.17)	0.19	(0.17)
His	0.42	(0.37)	0.29	(0.25)	0.29	(0.25)	0.29	(0.25)	0.29	(0.25)
Phe	0.79	(0.71)	0.53	(0.50)	0.53	(0.50)	0.53	(0.50)	0.53	(0.50)
Leu	1.37	(1.32)	0.95	(1.05)	0.95	(1.05)	0.95	(1.05)	0.95	(1.05)
Ile	0.72	(0.68)	0.67	(0.63)	0.67	(0.63)	0.67	(0.63)	0.67	(0.63)
Thr	0.69	(0.70)	0.69	(0.64)	0.69	(0.64)	0.69	(0.64)	0.69	(0.64)
Val	0.84	(0.84)	0.79	(0.78)	0.79	(0.78)	0.79	(0.78)	0.79	(0.78)
Gly	0.72	(0.63)	0.51	(0.44)	0.51	(0.44)	0.51	(0.44)	0.51	(0.44)
Tau	0.14	-	0.15	-	0.15	-	0.15	-	0.15	-
Ser	0.67	-	0.44	-	0.44	-	0.44	-	0.44	-
Glu	3.43	-	2.66	-	2.66	-	2.66	-	2.66	-
Pro	1.09	-	0.90	-	0.90	-	0.90	-	0.90	-
Ala	0.85	-	0.60	-	0.60	-	0.60	-	0.60	-
Tyr	0.52	-	0.33	-	0.33	-	0.33	-	0.33	-

GAA = guanidinoacetic acid; Cit = citrulline; CP = crude protein.

1 Values of all the amino acids presented were total amino acids (measured on an as-is basis). Values in brackets were calculated total amino acids.

2 SP: standard protein diet with 17% CP.

3 RP: reduced protein diet with 13% CP.

4 L-Arg, GAA and L-Cit were added on top of the RP diets at 0.35%, 0.46% and 0.35% in diets, RP-Arg, RP-GAA and RP-Cit, respectively, to the level of Arg in SP.

### 2.3 Analysis of serum mineral composition

The concentrations of Na, K, Ca, Mg, P, and Zn in the blood serum samples were measured in duplicate using the commercial kits in a Thermo Scientific Indiko Plus clinical chemistry analyzer (Thermo Fisher Scientific Inc., Waltham, MA, US) following the manufacturer's instruction. The kits used were Sodium (Na) Enzymatic Colorimetric Test (catalog number NA 3851, Randox Laboratories Ltd., County Antrim, UK), Potassium (K) U.V. Test (catalog number PT 3852, Randox Laboratories Ltd., County Antrim, UK), Calcium, Magnesium, and Phosphorus (Reference numbers 981772, 981905 and 981890 respectively, Thermo Fisher Scientific Inc., Waltham, MA, US). In addition, the blood serum Zn level was determined using a Zinc kit (catalog number ZN 2341, Randox Laboratories Ltd., County Antrim, UK) following the manufacturer's instruction, and the results were read on a SpectraMax M2e plate reader (Molecular Devices, California, USA).

### 2.4 Analysis of bone morphology and mineral composition in bone, feed, and excreta

Right leg bones including the tibia, femur, and middle toe were separated, cleaned, and dried in a fume hood for 48 h. Then bone weight, length, width, breaking strength, and ash content were measured according to Dao et al. (2022). Absolute tibia and femur ash content were calculated as a percentage of oven-dry bone (%). Relative tibia and femur ash per unit of BW were computed by dividing bone ash weight (g) to the hen BW (kg). Similarly, relative bone length, width, and breaking strength were calculated by dividing the absolute values measured on the air-dry bones to the hen BW. The mineral composition in the tibia, toe, feed, and excreta was quantified in an inductively coupled plasma-optical emission spectrometry instrument (Agilent, Victoria, Australia) following procedures previously described by Zanu et al. (2020).

### 2.5 Calculations and statistical analysis

Apparent mineral digestibility was calculated following equations described by Kong and Adeola (2014).

Apparent mineral digestibility (%) = (Mineral_retention_/Mineral_intake_) × 100;

Mineral_intake_ = Mineral_feed_ × Feed intake;

Mineral_retention_ = Mineral_intake_ – Mineral_excreta_ × Excreta output.

All data were calculated on a dry matter basis. Ingested Ca to P ratio was calculated by dividing Ca intake by P intake. In addition, mineral intake, excretion, and retention were computed as per unit of BW to remove the possible effect of growth rate on these variables.

Bone Seedor index (mg/mm) was calculated following an equation of Seedor et al. (1991).

$$Bone\;Seedor\;index\;\left(mg/mm\right)=\frac{Weight\;of\;oven\;dry\;bone\;\left(mg\right)}{Bone\;length\;\left(mm\right)}$$

R Commander (version 3.3.1, R Foundation for Statistical Computing, Vienna, Austria) was used to test statistical differences between the dietary treatments. Then, Tukey's post-hoc test was employed to identify pairwise differences between the treatments from significant ANOVA results. Additionally, possible correlations between the variables were tested using a Pearson's product–moment correlation test. *P*-values ≤0.05 were considered significant.

## 3 Results

### 3.1 Dietary mineral composition

The mineral composition of the dietary treatments is presented in Table 3. Generally, the mineral levels in the mixed diets satisfied formulation objectives and met the minimum requirements of the breed (Hy-Line International, 2016). Levels of minerals were similar between the diets except for K and B levels in the SP diet were higher than those of RP diets (Table 3).

**Table 3:** Analyzed mineral composition of experimental diets.

Mineral	SP^1^	RP^2^	RP-Arg^3^	RP-GAA^3^	RP-Cit^3^
Ca, mg/g	53.35	62.33	55.13	60.52	57.81
P, mg/g	8.24	7.66	7.68	7.87	7.73
Na, mg/g	2.03	2.15	1.99	2.35	2.13
Mg, mg/g	2.25	1.94	1.89	1.86	1.85
K, mg/g	8.41	4.80	4.80	4.82	4.94
S, mg/g	2.52	2.49	2.40	2.45	2.45
Al, μg/g	379	393	342	378	394
B, μg/g	9.05	2.60	2.36	2.51	2.60
Fe, μg/g	317	327	328	345	329
Cu, μg/g	11.66	13.18	10.78	12.64	12.20
Zn, μg/g	113	112	106	104	119
Mn, μg/g	95.94	95.36	103.64	105.42	87.95
Mo, μg/g	18.67	22.83	20.71	22.00	23.19

GAA = guanidinoacetic acid; Cit = citrulline.

1 SP: standard protein diet with 17% CP.

2 RP: reduced protein diet with 13% CP.

3 L-Arg, GAA and L-Cit were added on top of the RP diets at 0.35%, 0.46% and 0.35% in diets, RP-Arg, RP-GAA and RP-Cit, respectively, to the level of Arg in SP.

### 3.2 Bone morphology and strength

Results on the effects of dietary treatments on bone morphology and strength at wk 40 are presented in Table 4. Birds fed the SP and RP diet had similar absolute and relative bone weight, ash, length, width, Seedor index, and breaking strength (Table 4). Supplementation of Arg, GAA, and Cit to the RP diet increased relative femur weight (*P* < 0.001) and length (*P* < 0.001) compared to the SP treatment. Supplementation of both GAA and Cit to the RP diet increased relative tibia weight (*P* < 0.001), tibia length (*P* = 0.004), tibia width (*P* = 0.002), and femur width (*P* = 0.001) compared to the SP. Whereas, supplementation of Cit to the RP diet increased relative tibia ash (*P* = 0.030) and femur ash (*P* = 0.018) compared to the SP treatment. Feeding the RP-GAA diet increased relative femur length compared to the RP (*P* < 0.001, Table 4). Dietary treatments did not affect tibia and femur breaking strength and Seedor index, toe weight and ash at wk 40.

**Table 4:** Bone morphology and strength of laying hens in experimental treatments at wk 40.

Item	SP^1^	RP^2^	RP-Arg^3^	RP-GAA^3^	RP-Cit^3^	SEM	*P*-value
As-is basis							
Tibia weight^4^, g	9.13	9.24	8.98	9.13	9.11	0.110	0.961
Tibia ash^5^, %	40.70	40.01	39.10	37.43	39.11	0.551	0.422
Tibia length^6^, mm	125.2	123.0	122.6	123.1	122.4	0.51	0.451
Tibia Seedor index^7^, mg/mm	68.43	70.67	68.90	70.85	70.09	0.710	0.778
Tibia width, mm	7.90	7.95	7.73	7.92	7.86	0.047	0.659
Tibia breaking strength, N	269	281	225	249	244	7.9	0.130
Femur weight, g	6.81	7.01	7.02	6.89	7.01	0.106	0.967
Femur ash, %	47.15	46.85	45.61	45.05	46.05	0.737	0.902
Femur length, mm	87.92	87.04	87.97	87.27	87.09	0.359	0.876
Femur Seedor index, mg/mm	71.12	74.25	73.71	72.87	74.34	0.942	0.822
Femur width, mm	8.72	8.81	8.66	8.67	8.71	0.063	0.899
Femur breaking strength, N	313.9	299.8	256.5	300.2	287.8	14.49	0.787
Toe weight, g	3.18	3.02	2.85	2.82	2.80	0.068	0.333
Toe ash, %	13.86	14.58	14.82	14.91	14.44	0.150	0.172
As per unit of body weight							
Tibia weight, g/kg	4.01^a^	4.32^ab^	4.61^ab^	4.92^b^	4.84^b^	0.083	<0.001
Tibia ash, g/kg	1.63^a^	1.72^ab^	1.79^ab^	1.83^ab^	1.88^b^	0.027	0.030
Tibia length, mm/kg	58.80^a^	61.18^ab^	66.95^ab^	69.68^b^	69.48^b^	1.181	0.004
Tibia width, mm/kg	3.71^a^	3.95^ab^	4.23^ab^	4.54^b^	4.46^b^	0.090	0.002
Tibia breaking strength, N/kg	126.0	138.8	122.3	141.1	135.4	3.70	0.432
Femur weight, g/kg	2.93^a^	3.21^ab^	3.54^b^	3.63^b^	3.65^b^	0.067	<0.001
Femur ash, g/kg	1.38^a^	1.50^ab^	1.60^ab^	1.63^ab^	1.67^b^	0.032	0.018
Femur length, mm/kg	41.25^a^	43.30^ab^	48.09^bc^	49.99^c^	49.36^bc^	0.841	<0.001
Femur width, mm/kg	4.09^a^	4.38^ab^	4.73^ab^	4.96^b^	4.94^b^	0.091	0.001
Femur breaking strength, N/kg	146.8	148.4	137.5	167.2	157.4	6.57	0.688
Toe weight, g/kg	1.12	1.13	1.17	1.21	1.20	0.023	0.642
Toe ash, g/kg	0.15	0.17	0.17	0.18	0.18	0.004	0.327

GAA = guanidinoacetic acid; Cit = citrulline.

^a,b,c^Differing superscripts within a row indicate significant differences between means.

1 SP: standard protein diet with 17% CP.

2 RP: reduced protein diet with 13% CP.

3 L-Arg, GAA and L-Cit were added on top of the RP diets at 0.35%, 0.46% and 0.35% in diets, RP-Arg, RP-GAA and RP-Cit, respectively, to the level of Arg in SP.

4 Tibia, femur, and toe weight were measured on air-dry bones.

5 Absolute tibia and femur ash content were calculated as a percentage of oven-dry bone (%), relative tibia and femur ash per unit of body weight were computed by dividing bone ash weight (g) to the hen body weight (kg).

6 Bone length, width, and breaking strength were measured on air-dry bones.

7 Tibia and femur Seedor index were calculated by dividing the weight of oven-dry bone (mg) by bone length (mm).

### 3.3 Serum and bone mineral composition

The serum and bone mineral composition of the dietary treatments at wk 40 are shown in Table 5, Table 6. Levels of serum Ca, P, Na, Mg, K, and Zn were not different between the SP and RP diets (Table 5). Supplementation of GAA to the RP diet decreased serum Ca (*P* = 0.009), P (*P* = 0.009), and Mg (*P* < 0.001) levels compared to the SP (Table 5). Supplementation of either Arg or Cit to the RP diet did not affect serum mineral levels (Table 5). Interestingly, B was only detected in the tibia and toes of birds fed the SP diet (Table 6). Birds fed the SP diet tended to have lower Fe levels in the toes (*P* = 0.061) but similar Fe levels in the tibias compared to the RP-fed birds (Table 6). Supplementation of GAA to the RP diet decreased tibia Fe level (*P* = 0.016) and toe Mg level (*P* = 0.001) compared to the SP, and decreased tibia Mg level compared to the RP and RP-Arg diets (*P* = 0.022, Table 6). Supplementation of either Arg or Cit to the RP diet did not affect tibia and toe mineral composition at wk 40 (Table 6).

**Table 5:** Serum mineral composition of laying hens in experimental treatments at wk 40 (mg/dL).

Mineral	SP^1^	RP^2^	RP-Arg^3^	RP-GAA^3^	RP-Cit^3^	SEM	*P*-value
Ca	28.36^b^	22.48^ab^	22.37^ab^	18.73^a^	22.05^ab^	0.913	0.009
P	5.85^b^	4.92^ab^	5.06^ab^	3.77^a^	4.89^ab^	0.180	0.009
Na	395	398	398	399	399	1.7	0.938
Mg	3.82^b^	3.39^ab^	3.49^ab^	2.97^a^	3.38^ab^	0.074	<0.001
K	26.32	27.92	26.78	28.66	26.69	0.412	0.394
Zn	0.47	0.39	0.43	0.42	0.42	0.017	0.588

GAA = guanidinoacetic acid; Cit = citrulline.

^a,b^Differing superscripts within a row indicate significant differences between means.

1 SP: standard protein diet with 17% CP.

2 RP: reduced protein diet with 13% CP.

3 L-Arg, GAA and L-Cit were added on top of the RP diets at 0.35%, 0.46% and 0.35% in diets, RP-Arg, RP-GAA and RP-Cit, respectively, to the level of Arg in SP.

**Table 6:** Tibia and toe mineral composition of laying hens in experimental treatments at wk 40.

Mineral	SP^1^	RP^2^	RP-Arg^3^	RP-GAA^3^	RP-Cit^3^	SEM	*P*-value
Tibia							
Ca, mg/g	397	407	401	405	400	1.9	0.482
P, mg/g	174	180	178	178	177	1.1	0.564
Na, mg/g	10.49	10.56	10.44	10.54	10.35	0.056	0.764
Mg, mg/g	5.86^ab^	5.87^b^	5.87^b^	5.51^a^	5.74^ab^	0.042	0.022
K, mg/g	3.71	3.60	3.38	3.47	3.39	0.069	0.485
S, mg/g	1.41	1.36	1.27	1.33	1.37	0.022	0.306
Al, μg/g	4.15	9.06	7.82	8.04	8.29	0.683	0.176
B, μg/g	0.31	0.00	0.00	0.00	0.00	0.044	0.086
Fe, μg/g	156^b^	140^ab^	132^ab^	113^a^	126^ab^	4.3	0.016
Cu, μg/g	0.13	0.62	0.36	0.64	0.20	0.116	0.430
Zn, μg/g	407	412	421	394	429	5.3	0.288
Mn, μg/g	11.63	12.81	10.53	10.58	11.81	0.561	0.697
Mo, μg/g	59.41	86.23	72.61	77.66	72.38	4.463	0.753
Toe							
Ca, mg/g	405	401	403	403	403	0.8	0.638
P, mg/g	180	177	178	176	177	0.5	0.306
Na, mg/g	17.88	17.90	18.01	18.21	18.16	0.132	0.841
Mg, mg/g	4.87^b^	4.74^ab^	4.75^ab^	4.55^a^	4.69^ab^	0.026	0.001
K, mg/g	6.37	6.29	6.23	6.11	6.26	0.092	0.929
S, mg/g	2.83	2.68	2.75	2.69	2.82	0.049	0.904
Al, μg/g	109.0	118.0	107.0	100.0	92.4	5.01	0.579
B, μg/g	2.13^b^	0.00^a^	0.10^a^	0.00^a^	0.00^a^	0.133	<0.001
Fe, μg/g	136	188	182	173	143	7.1	0.061
Cu, μg/g	5.21	5.06	4.54	3.49	4.91	0.252	0.185
Zn, μg/g	324	325	330	321	326	3.0	0.911
Mn, μg/g	15.34	14.63	13.52	14.27	14.64	0.253	0.240
Mo, μg/g	114	114	116	115	112	0.5	0.198

GAA = guanidinoacetic acid; Cit = citrulline.

^a,b^Differing superscripts within a row indicate significant differences between means.

1 SP: standard protein diet with 17% CP.

2 RP: reduced protein diet with 13% CP.

3 L-Arg, GAA and L-Cit were added on top of the RP diets at 0.35%, 0.46% and 0.35% in diets, RP-Arg, RP-GAA and RP-Cit, respectively, to the level of Arg in SP.

### 3.4 Fat and fiber intake and apparent mineral digestibility

Results on fat, fiber and mineral intake are presented in Table 7. Results on mineral excretion, retention, and digestibility of experimental treatments at wk 40 are shown in Table 8, Table 9, Table 10, respectively. Birds fed the SP diets had higher fat intake (*P* < 0.001) but lower fiber intake (*P* < 0.001) and lower ingested Ca:P ratio (*P* < 0.001) compared to birds offered the RP, RP-Arg, RP-GAA, and RP-Cit diets (Table 7). Birds fed the SP ingested more K (*P* < 0.01) and B (*P* < 0.01) compared to those fed the RP, RP-Arg, RP-GAA, and RP-Cit diets (Table 7). Supplementation of GAA to the RP diet decreased Mg intake (*P* = 0.008) and tended to decrease P (*P* = 0.089) and Zn intake (*P* = 0.051) compared to those offered SP diets (Table 7). A similar trend was observed when mineral intake was expressed as per unit of BW although relative P and Zn intake were not significantly different between the dietary treatments (Table 7).

**Table 7:** Mineral, fat and fiber intake of laying hens in experimental treatments at wk 40.

Item	SP^1^	RP^2^	RP-Arg^3^	RP-GAA^3^	RP-Cit^3^	SEM	*P*-value
As-is basis (mg/d for minerals and g/d for fat and fiber)							
Ca	8333	7693	7795	6698	8113	284.4	0.452
P	1287	1040	1086	871	1084	47.4	0.089
Na	317	292	282	260	299	11.4	0.651
Mg	352^b^	263^ab^	267^ab^	206^a^	259^ab^	13.6	0.008
K	1314^b^	652^a^	678^a^	533^a^	694^a^	62.2	0.003
S	394	337	340	271	343	14.6	0.128
Al	59.17	53.41	48.41	41.87	55.31	2.243	0.137
B	1.41^b^	0.35^a^	0.33^a^	0.28^a^	0.36^a^	0.086	0.002
Fe	49.48	44.44	46.36	38.19	46.17	1.812	0.419
Cu	1.82	1.63	1.52	1.40	1.71	0.061	0.241
Zn	17.57	15.21	14.98	11.47	16.73	0.693	0.051
Mn	14.99	12.95	14.65	11.67	12.34	0.551	0.256
Mo	2.92	3.10	2.93	2.44	3.25	0.120	0.301
Fat	5.86^b^	3.47^a^	3.39^a^	3.47^a^	3.29^a^	0.186	<0.001
Fiber	5.99^a^	11.35^b^	11.08^b^	11.34^b^	10.73^b^	0.475	<0.001
As per unit of body weight (mg/d per kg body weight for minerals and g/d per kg body weight for fat and fiber)							
Ca	4143	4508	4042	3588	4250	179.2	0.632
P	640	554	563	467	568	25.4	0.351
Na	157	156	146	139	157	6.5	0.901
Mg	175	140	138	110	136	7.0	0.059
K	653^b^	347^a^	352^a^	286^a^	364^a^	30.9	0.005
S	196	180	176	145	180	7.9	0.394
Al	29.42	28.46	25.10	22.43	28.97	1.221	0.340
B	0.70^b^	0.19^a^	0.17^a^	0.15^a^	0.19^a^	0.043	0.003
Fe	24.60	23.67	24.04	20.46	24.19	1.011	0.758
Cu	0.91	0.95	0.79	0.75	0.90	0.042	0.459
Zn	8.74	8.10	7.77	6.14	8.77	0.374	0.166
Mn	7.45	6.90	7.60	6.25	6.47	0.310	0.615
Mo	1.45	1.65	1.52	1.30	1.71	0.073	0.377
Fat	2.75^b^	1.73^a^	1.84^a^	1.95^a^	1.83^a^	0.082	<0.001
Fiber	2.81^a^	5.64^b^	6.02^b^	6.38^b^	5.96^b^	0.266	<0.001
Ingested Ca:P	6.48^a^	7.88^e^	7.18^b^	7.69^d^	7.48^c^	0.081	<0.001

GAA = guanidinoacetic acid; Cit = citrulline.

^a–e^Differing superscripts within a row indicate significant differences between means.

1 SP: standard protein diet with 17% CP.

2 RP: reduced protein diet with 13% CP.

3 L-Arg, GAA and L-Cit were added on top of the RP diets at 0.35%, 0.46% and 0.35% in diets, RP-Arg, RP-GAA and RP-Cit, respectively, to the level of Arg in SP.

**Table 8:** Mineral excretion of laying hens in experimental treatments at wk 40.

Mineral	SP^1^	RP^2^	RP-Arg^3^	RP-GAA^3^	RP-Cit^3^	SEM	*P*-value
As-is basis (mg/d)							
Ca	2242	1751	1679	1314	1894	127.8	0.221
P	570^b^	477^ab^	504^ab^	346^a^	477^ab^	22.1	0.013
Na	88.95	64.86	67.20	46.47	65.00	6.011	0.332
Mg	154^b^	122^ab^	130^ab^	93.2^a^	128^ab^	5.7	0.024
K	605^b^	329^a^	364^a^	284^a^	345^a^	25.9	0.009
S	119^b^	94.10^ab^	92.47^ab^	72.97^a^	96.01^ab^	4.449	0.012
Al	27.95^b^	23.12^ab^	23.61^ab^	15.53^a^	21.42^ab^	1.259	0.019
B	0.74^b^	0.22^a^	0.24^a^	0.17^a^	0.23^a^	0.043	0.001
Fe	25.10^b^	19.11^ab^	19.40^ab^	13.24^a^	20.35^ab^	1.150	0.013
Cu	1.16^b^	0.74^a^	0.77^ab^	0.53^a^	0.81^ab^	0.055	0.027
Zn	9.56^b^	7.47^ab^	7.39^ab^	5.28^a^	7.47^ab^	0.412	0.013
Mn	7.43^b^	6.16^ab^	6.61^ab^	4.69^a^	6.44^ab^	0.292	0.034
Mo	0.63^b^	0.44^ab^	0.45^ab^	0.36^a^	0.48^ab^	0.026	0.009
As per unit of body weight (mg/d per kg body weight)							
Ca	1087	938	870	702	1008	67.4	0.443
P	282	257	262	183	248	12.3	0.094
Na	43.46	33.62	34.80	24.99	34.96	3.057	0.457
Mg	76.37	65.84	67.69	49.56	67.26	3.261	0.106
K	299^b^	177^a^	189^a^	150^a^	181^a^	13.3	0.007
S	58.67	50.42	48.12	38.73	50.42	2.430	0.120
Al	13.73	12.45	12.26	8.22	11.26	0.664	0.076
B	0.36^b^	0.12^a^	0.12^a^	0.09^a^	0.12^a^	0.021	0.002
Fe	12.37	10.28	10.03	7.05	10.78	0.619	0.075
Cu	0.57^b^	0.40^ab^	0.40^ab^	0.28^a^	0.42^ab^	0.027	0.030
Zn	4.69^b^	3.99^ab^	3.82^ab^	2.80^a^	3.89^ab^	0.205	0.047
Mn	3.65	3.32	3.42	2.49	3.39	0.161	0.173
Mo	0.31	0.24	0.24	0.19	0.25	0.014	0.079

GAA = guanidinoacetic acid; Cit = citrulline.

^a,b^Differing superscripts within a row indicate significant differences between means.

1 SP: standard protein diet with 17% CP.

2 RP: reduced protein diet with 13% CP.

3 L-Arg, GAA and L-Cit were added on top of the RP diets at 0.35%, 0.46% and 0.35% in diets, RP-Arg, RP-GAA and RP-Cit, respectively, to the level of Arg in SP.

**Table 9:** Mineral retention of laying hens in experimental treatments at wk 40.

Mineral	SP^1^	RP^2^	RP-Arg^3^	RP-GAA^3^	RP-Cit^3^	SEM	*P*-value
As-is basis (mg/d)							
Ca	5324	6104	6116	5342	6219	265.3	0.718
P	598	563	582	551	607	35.0	0.988
Na	198	228	214	208	234	10.4	0.834
Mg	140	141	137	117	131	8.2	0.924
K	493	323	314	253	349	25.9	0.135
S	242	243	247	200	247	11.8	0.720
Al	24.99	30.30	24.79	26.78	33.88	1.853	0.478
B	0.67^b^	0.13^a^	0.10^a^	0.12^a^	0.13^a^	0.055	0.007
Fe	18.98	25.33	26.97	25.57	25.82	1.642	0.624
Cu	0.44	0.93	0.76	0.89	0.91	0.064	0.073
Zn	6.16	7.73	7.60	6.39	9.27	0.502	0.278
Mn	6.00	6.78	8.04	7.18	5.90	0.434	0.528
Mo	2.29	2.66	2.48	2.07	2.77	0.121	0.370
As per unit of body weight (mg/d per kg body weight)							
Ca	2616	3393	3172	2850	3242	155.4	0.552
P	290	297	301	291	320	17.6	0.987
Na	97	122	111	111	122	5.8	0.681
Mg	69.34	74.21	70.76	61.84	68.48	4.013	0.919
K	244	171	162	134	182	12.5	0.126
S	118	129	128	106	129	6.1	0.750
Al	12.23	16.01	12.84	14.25	17.71	0.951	0.344
B	0.34^b^	0.07^a^	0.05^a^	0.06^a^	0.07^a^	0.028	0.007
Fe	9.24	13.40	14.01	13.55	13.41	0.852	0.424
Cu	0.22^a^	0.52^b^	0.39^ab^	0.47^ab^	0.48^ab^	0.034	0.045
Zn	4.04	4.11	3.95	4.26	4.88	0.262	0.193
Mn	2.93	3.58	4.18	3.81	3.08	0.220	0.395
Mo	1.14	1.41	1.28	1.11	1.45	0.072	0.188

GAA = guanidinoacetic acid; Cit = citrulline.

^a,b^Differing superscripts within a row indicate significant differences between means.

1 SP: standard protein diet with 17% CP.

2 RP: reduced protein diet with 13% CP.

3 L-Arg, GAA and L-Cit were added on top of the RP diets at 0.35%, 0.46% and 0.35% in diets, RP-Arg, RP-GAA and RP-Cit, respectively, to the level of Arg in SP.

**Table 10:** Apparent mineral digestibility of laying hens in experimental treatments at wk 40 (%).

Mineral	SP^1^	RP^2^	RP-Arg^3^	RP-GAA^3^	RP-Cit^3^	SEM	*P*-value
Ca	65.95	79.53	78.38	80.10	75.76	1.742	0.453
P	47.35	53.05	52.95	63.53	55.21	1.821	0.089
Na	64.51	78.24	76.38	79.97	77.51	2.040	0.498
Mg	44.02	52.24	50.68	57.60	49.35	1.631	0.168
K	41.42	48.04	45.84	48.07	49.31	1.392	0.510
S	63.57	71.53	72.36	74.08	71.42	1.193	0.054
Al	43.06^a^	56.17^ab^	50.82^ab^	64.05^b^	60.13^ab^	2.223	0.021
B	44.38	35.94	27.88	42.91	35.05	2.514	0.235
Fe	38.20^a^	56.71^ab^	57.48^ab^	67.15^b^	54.47^ab^	2.631	0.037
Cu	23.25^a^	58.08^b^	49.37^b^	63.99^b^	51.85^b^	3.432	0.010
Zn	34.89^a^	50.61^ab^	50.44^ab^	55.73^b^	54.93^b^	2.301	0.023
Mn	40.57^a^	51.42^ab^	54.80^ab^	61.83^b^	46.71^ab^	2.040	0.009
Mo	76.79	85.50	84.37	85.13	84.74	1.113	0.194

GAA = guanidinoacetic acid; Cit = citrulline.

^a,b^Differing superscripts within a row indicate significant differences between means.

1 SP: standard protein diet with 17% CP.

2 RP: reduced protein diet with 13% CP.

3 L-Arg, GAA and L-Cit were added on top of the RP diets at 0.35%, 0.46% and 0.35% in diets, RP-Arg, RP-GAA and RP-Cit, respectively, to the level of Arg in SP.

Higher K (*P* = 0.009), B (*P* = 0.001), and Cu (*P* = 0.027) excretion were observed in birds fed the SP diet compared to those offered the RP diet (Table 8). Supplementation of GAA to the RP diet reduced P (*P* = 0.013), Mg (*P* = 0.024), S (*P* = 0.012), Al (*P* = 0.019), Fe (*P* = 0.013), Zn (*P* = 0.013), Mn (*P* = 0.034), and Mo (*P* = 0.009) excretion compared to the SP diet (Table 8). Similar trends were observed when mineral excretion was expressed as relative to BW although relative Mg, S, and Mn excretion were not significantly different between the treatment groups, and levels of excreted P (*P* = 0.094), Al (*P* = 0.076), Fe (*P* = 0.075), and Mo (*P* = 0.079) were only marginally affected by the dietary treatments (Table 8).

Birds fed the SP diet retained higher absolute and relative B (*P* = 0.007) compared to those fed the RP diet (Table 9). Whereas, birds fed the RP diets tended to retain higher absolute Cu (*P* = 0.073) and higher relative Cu (*P* = 0.045) compared to the SP-fed birds (Table 9). Thus, apparent Cu digestibility was higher in birds fed the RP compared to the SP diet (*P* = 0.010, Table 10). Supplementation of GAA to the RP diet increased Al (*P* = 0.021), Fe (*P* = 0.037), Zn (*P* = 0.023), and Mn (*P* = 0.009) digestibility and tended to increase P (*P* = 0.089), and S (*P* = 0.054) digestibility compared to the SP treatment (Table 10). Supplementation of Cit to the RP diet increased Zn digestibility compared to the SP treatment (*P* = 0.023) while supplementation of Arg to the RP diet did not affect mineral digestibility (Table 10).

### 3.5 Correlations between the variables

Correlations between the bone ash content, bone mineral level, serum mineral level and nutrient intake, excretion, and retention are presented in Table 11. Positive correlations between the tibia and femur ash and fiber intake were observed (*P* < 0.01, Table 11). Tibia and toe B levels were positively correlated to the level of B intake (*P* < 0.05, Table 11). Also, B intake was positively correlated to B retention and K intake was positively correlated to K excretion (*P* < 0.001, Table 11). Whereas, ingested Ca to P ratio was negatively correlated to serum Ca and P levels (*P* < 0.001, Table 11).

**Table 11:** Correlations between measured parameters.

Parameter	Correlation coefficient (*r*)	*P*-value
Fiber intake – femur ash	0.425	0.002
Fiber intake – tibia ash	0.408	0.004
B intake – tibia B	0.312	0.028
B intake – toe B	0.605	<0.001
B intake – B retention	0.834	<0.001
K intake – K excretion	0.686	<0.001
Ingested Ca:P – serum Ca	−0.517	<0.001
Ingested Ca:P – serum P	−0.540	<0.001
Zn intake – tibia Zn	0.273	0.055
Fat intake – femur ash	−0.236	0.102
Fat intake – tibia ash	−0.197	0.175

## 4 Discussion

Increasing dietary Arg supplementation in broilers has been reported to increase tibia and femur Seedor index, femur length, and tibia diameter (Fernandes et al., 2014). Furthermore, Arg supplementation above the requirement for BW gain in broilers could reduce the proportion of broken wings during processing (Corzo et al., 2003). In the current study, there were no differences in bone weight, ash, length, width, Seedor index, and breaking strength between birds fed the SP and RP diets. However, supplementation of Arg, GAA, or Cit to the RP diet were all effective in increasing bone morphology in birds. More positive effects on bone morphology were observed with GAA and Cit supplementation compared to Arg. Besides, Cit supplementation increased relative tibia and femur ash compared to birds offered the SP diet in the current study. Previous studies have demonstrated that crystalline amino acids such as Arg are more digestible than protein bound-amino acids (Hilliar et al., 2019) and Cit supplementation resulted in higher blood Arg levels compared to dietary Arg (Lassala et al., 2009; Schwedhelm et al., 2008). Additionally, it has been indicated that reducing dietary Zn levels may not only reduce growth but can also shorten long bones, impair bone mineralization, and increase skeletal malformations (Kidd et al., 1996; Naz et al., 2016). In the present study, the higher Zn digestibility observed in the RP-Cit treatment might increase bone ash compared to those in the SP treatment. This may be expected as higher dietary fiber levels have been shown to increase intestinal mineral absorption (Gultemirian et al., 2014). Positive correlations between the tibia and femur ash and fiber intake were observed in the current study. It is possible that the higher fiber intake in hens fed the RP-Cit diet increased intestinal mineral absorption resulting in increased bone ash compared to those offered the SP diet. Osteoporosis has been reported as the main cause of mortality in laying hens housed under the cage systems (Mayeda and Ernst, 2008). Dietary supplementation of Arg, GAA, or Cit can effectively improve bird health and welfare and reduce the economic loss for egg producers.

Effects of reducing dietary CP level and/or amino acid imbalance on blood and bone mineral composition in birds have been often overlooked. Available data has shown that broiler chickens fed reduced CP diets (21% vs. 24% CP from d 0 to 28, and 18% vs. 21% CP from d 29 to 56) had higher serum Ca, Na, and Zn levels compared to those offered standard CP diets (Pisarski, 1988). Likewise, Cowieson et al. (2020) reported increased plasma Ca level in broiler chickens when dietary CP level decreased from 21.5% and 19.5% to 17.5% and 15.5% in the grower and finisher phase, respectively. In breeder males, Mahmood et al. (2016) observed that feeding a reduced CP diet with 12% CP increased serum Ca, Na, Mg, and Zn levels, but decrease serum K level compared to a normal protein diet with 16% CP. It has been suggested that the blood-Ca-increasing effect of low CP diets may be associated with decreased Ca reabsorption as a result of the hypo-calciuretic effect following an alteration of the kidney function (Cowieson et al., 2020; Linkswiler et al., 1981). The results of the current study indicated that hens fed the Arg-deficient RP diet had similar levels of all examined serum minerals (Ca, P, Na, Mg, K, and Zn) compared to the hens offered the SP diet at wk 40. Also, supplementation of either Arg or Cit to the Arg-deficient RP diet did not affect serum mineral composition while supplementation of GAA to the RP diet decreased serum Ca, P, and Mg levels compared to the SP. These results suggest the differential effects of reducing dietary CP levels on serum mineral levels in broilers and laying hens. Serum mineral levels of laying hens were not affected by dietary protein level, Arg deficiency, or amino acid imbalance in the current study. Previous studies have indicated that mineral utilization in birds is prioritized for maintaining circulating mineral levels (essential functions) rather than growth/production purposes (Hosseini and Afshar, 2017). The lower serum Ca, P, and Mg levels in birds fed the RP-GAA diet in the current study were more likely due to the lower intake of these minerals in the respective group compared to those fed the SP diet despite the birds efforts to increase the mineral digestibility. Reducing dietary Ca, P, and/or Mg levels have been reported to decrease levels of these minerals in the blood serum (Atteh and Leeson, 1983; Jiang et al., 2013; Jing et al., 2018). The low serum Ca and P levels in birds fed the RP-GAA diet may reflect the low metabolism of the whole body Ca and P (Veum, 2010). Additionally, the lower serum Ca and P levels in birds fed the RP-GAA diet in the current study might be caused by a higher ingested Ca to P ratio in this group compared to that of the SP-fed birds. There is evidence that reducing the dietary Ca to P ratios could increase serum Ca and P levels in broilers (Bilal et al., 2015). The results of the current study supported this as negative correlations were observed between ingested Ca to P ratio and levels of Ca and P in the serum.

Dietary protein and Arg both affect bone mineral concentration in birds. Castro et al. (2019a) observed decreased bone mineral density in broilers fed an Arg-deficient diet. Cowieson et al. (2020) reported that feeding RP diets (17.5% vs. 21.5% and 15.5% vs. 19.5% CP in the grower and finisher phase, respectively) decreased tibia Mn and Cu levels of broiler chickens compared to the SP diets. In the current study, lower tibia and toe B levels in birds fed the RP diet were attributed to the lower B intake and retention in the respective group compared to those offered the SP diet, as shown by the results of the correlation tests. The current findings were supported by previous studies, which showed increased tibia B levels as dietary B levels increase (Kurtoğlu et al., 2005; Olgun and Bahtiyarca, 2015). Besides, the results of the current study indicated that supplementation of either Arg or Cit to the RP diet did not affect bone mineral levels while supplementation of GAA to the RP diet decreased tibia Mg level compared to the RP, and decreased tibia Fe and toe Mg levels compared to the SP. These findings were consistent with the mineral intake and digestibility results in the present study. The current findings were supported by Atteh and Leeson (1983) who found increased plasma and tibia Mg levels in laying hens as dietary Mg levels increased (from 0.17% to 0.77%). No effects on feed intake, egg production, shell weight, shell thickness, and internal egg quality were reported by Dao et al. (2021b) when either Arg or Cit was supplemented to an Arg-deficient RP diet for laying hens. The lack of Arg and Cit effects on serum and bone mineral level and mineral digestibility in the current study might be due to the similar mineral intakes resulting from similar feed intakes in birds fed the RP-Arg and RP-Cit diets compared to those fed the RP diet as noted by Dao et al. (2021b). Additionally, the lower dietary electrolyte balance levels in the RP compared to the SP diets may decrease bone integrity (Araujo et al., 2022) and limit the effects of Arg and Cit supplementation in the RP diets in the current study. Determining the effects of dietary electrolyte balance in laying hens fed reduced-protein diets may be worthwhile for further studies.

One important finding of the current study is the difference in response to individual minerals in the diet. Increasing dietary B resulted in higher B retention and higher tibia and toe B levels as observed in birds fed the SP diet compared to those fed the RP diet. Excess dietary K increased K excretion with no effect on K retention in the SP-fed birds. A positive correlation was observed between levels of K intake and excretion in the current study. These results suggest that consumption of B is more important than K or B is more digestible than K in the SP-fed birds. The mineral digestibility results in the current study showed that B and K digestibility in the SP-fed birds were 44.38% and 41.42%, respectively. In contrast, lower B digestibility compared to K digestibility was observed in all RP diets in the current study. On average, B and K digestibility of birds fed the RP, RP-Arg, RP-GAA, and RP-Cit diet at wk 40 were 35.44% and 47.82%, respectively. These findings suggest possible interactions between dietary protein level and mineral digestibility in laying hens.

In the current study, birds fed the RP-GAA diet responded to decreased Mg, P, and Zn intake by reducing the excretion of these minerals. In addition, decreased excretion of S, Al, Fe, Mn, and Mo and increased digestibility of Al, Fe, Zn, Mn, P, and S were observed in birds fed the RP-GAA diet compared to those fed the SP diet in the current study. An adaptation capacity involving complex hormonal mechanisms of Ca homeostasis, to increase Ca utilization, was observed in broilers fed diets deficient in Ca (Mansilla et al., 2020; Proszkowiec-Weglarz and Angel, 2013; Yan et al., 2005). Also, there is evidence that additional supplementation of trace minerals above the recommended levels did not improve growth performance and immunity status in birds and even led to a marked increase in mineral excretion (Güçlü et al., 2008; Skrivan et al., 2006; Yang et al., 2011). The capacity to sustain serum and bone trace mineral levels as well as bone morphology and strength in birds fed the RP-GAA diet in the current study may provide important information for a better understanding of trace mineral digestibility in birds.

## 5 Conclusions

Bone morphology, bone breaking strength and serum mineral level in laying hens were not influenced by dietary CP levels. Supplementation of Arg, GAA, or Cit to the RP diet was effective in increasing bone weight and length. The current study demonstrated the capacity of laying hens to adapt to low mineral intake by increasing the utilization of minerals as observed in the RP-GAA treatment. Analysis of egg mineral composition is warranted to comprehensively evaluate mineralization status in laying hens fed RP diets.

## Author contributions

**Hiep Thi Dao**: conceptualization, methodology, formal analysis, validation, writing original draft, review, statistics and editing. **Amy F. Moss**: methodology, review and editing. **Emma J. Bradbury**: review and editing. **Robert A. Swick**: methodology, review, editing and validation.

## Declaration of competing interest

We declare that we have no financial and personal relationships with other people or organizations that can inappropriately influence our work, and there is no professional or other personal interest of any nature or kind in any product, service and/or company that could be construed as influencing the content of this paper.
